# Nanostructured fibrin agarose hydrogel as a novel haemostatic agent

**DOI:** 10.1002/term.2831

**Published:** 2019-03-20

**Authors:** Rafael Campos‐Cuerva, Beatriz Fernández‐Muñoz, Francisco Farfán López, Sheila Pereira Arenas, Mónica Santos‐González, Luis Lopez‐Navas, Miguel Alaminos, Antonio Campos, Jordi Muntané, Carmen Cepeda‐Franco, Miguel Ángel Gómez‐Bravo

**Affiliations:** ^1^ Centro de Transfusiones, Tejidos y Células de Sevilla (CTTS) Seville Spain; ^2^ Cell Therapy and Cell Reprogramming Unit, GMP Network of the Andalusian Initiative for Advanced Therapies Junta de Andalucia Seville Spain; ^3^ PhD Program in Molecular Biology, Biomedicine and Clinical Research Universidad de Sevilla 41013 Seville Spain; ^4^ Instituto de Biomedicina de Sevilla IBIS (HUVR/CSIC/Universidad de Sevilla, Junta de Andalucía) Spain; ^5^ Servicio de Anatomía Patológica Hospital Universitario Virgen del Rocío (HUVR) Seville Spain; ^6^ Andalusian Initiative for Advanced Therapies Junta de Andalucia Seville Spain; ^7^ Department of Histology University of Granada Granada Spain; ^8^ Instituto de Investigación Biosanitaria ibs.GRANADA, 18016 Granada Spain; ^9^ Centro de Investigación Biomédica en red de Enfermedades Hepáticas y Digestivas (CIBEREHD) Madrid Spain; ^10^ Unidad de Cirugia Hepato‐Bilio‐Pancreática y Trasplantes HUVR Spain

**Keywords:** bleeding, fibrin agarose hydrogel, fibrin sealant, haemostasis, haemostatic agent, liver resection, nanostructured biomaterial, surgery

## Abstract

Blood loss remains a major concern during surgery and can increase the morbidity of the intervention. The use of topical haemostatic agents to overcome this issue therefore becomes necessary. Fibrin sealants are promising haemostatic agents due to their capacity to promote coagulation, but their effectiveness and applicability need to be improved. We have compared the haemostatic efficacy of a novel nanostructured fibrin‐agarose hydrogel patch, with (c‐NFAH) or without cells (a‐NFAH), against two commercially available haemostatic agents in a rat model of hepatic resection. Hepatic resections were performed by making short or long incisions (mild or severe model, respectively), and haemostatic agents were applied to evaluate time to haemostasis, presence of haematoma, post‐operative adhesions to adjacent tissues, and inflammation factors. We found a significantly higher haemostatic success rate (time to haemostasis) with a‐NFAH than with other commercial haemostatic agents. Furthermore, other relevant outcomes investigated were also improved in the a‐NFAH group, including no presence of haematoma, lower adhesions, and lower grades of haemorrhage, inflammation, and necrosis in histological analysis. Overall, these findings identify a‐NFAH as a promising haemostatic agent in liver resection and likely in a range of surgical procedures.

## 1 INTRODUCTION

Uncontrolled haemorrhage remains an important intraoperative complication that may change patient follow‐up after major surgical interventions and is responsible for high rates of mortality and morbidity and longer hospitalization duration (Brustia, Granger, & Scatton, 2016). In particular, hepatic resection often requires perioperative transfusion due to intraoperative blood loss. Despite improvements in existing surgical, anaesthesia preassessment and/or other perioperative interventions (suturing, cauterization, patient positioning, and use of special equipment such as water jet, radiofrequency ablation, or cavitron ultrasonic surgical aspirator), the reduction of bleeding during hepatic surgery remains transitory or incomplete (Berrevoet & Hemptinne, 2007; Brustia et al., 2016; Chouillard, Gumbs, & Cherqui, 2010; Eeson & Karanicolas, 2016; Smyrniotis, Farantos, Kostopanagiotou, & Arkadopoulos, 2005; Tympa et al., 2012; Zhu et al., 2015). Accordingly, topical haemostatic agents are usually required to prevent intraoperative or post‐operative blood loss (Eeson & Karanicolas, 2016; Moggia et al., 2016).

Several haemostatic agents approved by Food and Drug Administration have been developed to maintain haemostasis in multiple surgical procedures. Topical haemostatic agents can be classified according to their composition in matrix‐based, fibrin and/or thrombin‐based and combined products (matrix + fibrin and/or thrombin‐based haemostatic agent). Matrix‐based products form a barrier that interrupt blood flow facilitating clot formation (e.g., Gelfoam, Hemopatch) whereas fibrin and/or thrombin‐based products provide factors and compounds that promote generation of fibrin clots (e.g., Tissucol, Evicel). Combined products (e.g., Tachosil, Evarrest) seem to achieve better results (Brustia et al., 2016).

Fibrin sealant derived from human plasma has become a popular haemostatic agent due to its capacity to imitate and promote the final step of the coagulation cascade (Mankad & Codispoti, 2001; MOSESSON, SIEBENLIST, & MEH, 2006). Furthermore, fibrin sealant is the only Food and Drug Administration clinical material approved as a haemostatic sealant and adhesive agent for therapeutic use. Despite recent advances in the field, however, overall haemostatic suitability in terms of effectiveness (time to haemostasis), applicability, or absence of rebleeding or undesired adhesion remains far from perfect and could be further improved (Spotnitz, 2014).

We have previously developed a nanostructured fibrin and type VII agarose hydrogel (NFAH) as a scaffolding biomaterial compatible with multiple tissue models (Alaminos et al., 2006; Carriel et al., 2012, 2013; Carriel, Garzón, Campos, Cornelissen, & Alaminos, 2014; Garzón et al., 2014; Sanchez‐Quevedo et al., 2007). This biomaterial can be seeded with different cell types and has been characterized as a substitute for cornea, oral mucosa, and nerve (Carriel et al., 2017; Ionescu et al., 2011; Rodríguez et al., 2012). This stromal‐like substitute is composed mainly of human plasma and shows high flexible and elastic properties as well as mechanical strength (Mosesson et al., 2006; Scionti et al., 2014). Moreover, fibroblasts and other cell types can be seeded inside the scaffold with good viability and function (Carriel et al., 2013).

In the present study, we explored the haemostatic effectiveness of NFAH, with and without seeded allogeneic fibroblasts, in a partial hepatectomy rat model of short or long hepatic incision (mild or severe hepatic resection, respectively). The performance of the acellular (a‐NFAH) or cellularized (c‐NFAH) scaffold was compared with two commercially available haemostatic products commonly used in multiple surgical procedures: a polyethylene glycol‐coated collagen pad (PCC, Hemopatch®; Lewis, Ikeme, Olubunmi, & Kuntze, 2018) and a fibrinogen/thrombin‐coated collagen pad (FTC, Tachosil®; Rickenbacher, Breitenstein, Lesurtel, & Frilling, 2009).

## 2 MATERIALS AND METHODS

### 2.1 Cell culture

Fibroblasts used in this study were isolated from Wistar rat dermal tissue. Skin biopsies were thoroughly washed in phosphate‐buffered saline (PBS) containing penicillin–streptomycin solution (Sigma‐Aldrich, St. Louis, MO, USA), 20 μg/ml gentamicin, and 50 μg/ml vancomycin (Normon Laboratories S.A., Madrid, Spain) and cut into pieces of approximately 1 × 1 mm^2^. Twenty pieces were transferred to a 50‐ml conical centrifuge tube containing 5 ml of preheated 2‐mg/ml collagenase solution (SERVA Electrophoresis GmbH, Heidelberg, Germany) prepared in Dulbecco's modified Eagle's medium (Sigma‐Aldrich). Samples were incubated on rotation for 16 hr at 37°C. Digestion was neutralized by addition of Dulbecco's modified Eagle's medium supplemented with 10% fetal bovine serum (Sigma‐Aldrich, St. Louis, MO), 0.1‐mM non‐essential amino acids (Sigma‐Aldrich), 2‐mM Glutamax (Gibco, Fisher Scientific, Pittsburgh, PA, USA), and penicillin–streptomycin solution. Finally, the cell suspension was pelleted, counted, plated in a 25‐cm^2^ flask, and grown in a humidified incubator at 5% CO_2_ and 37°C.

Culture medium was changed every 2–3 days until the culture reached 70–90% confluence. At this point, cells were harvested and expanded until Passage 2. Finally, cells were cryopreserved in liquid nitrogen until generation of the hydrogels.

### 2.2 Immunofluorescence

Fibroblasts were seeded onto glass coverslips (VWR, Radnor, PA, USA) in 24‐well plates (Nalgene Nunc International, Rochester, NY, USA) and incubated in a humidified incubator with 5% CO_2_ at 37°C. Once confluent, coverslips were washed with PBS and fixed with 3.7% formaldehyde (Sigma‐Aldrich) in PBS for 15 min at room temperature. The coverslips were then washed three times with PBS, and cells were permeabilized with 0.05% Triton X‐100 (Sigma‐Aldrich) for 15 min at room temperature and blocked with PBS containing 1% bovine serum albumin (Sigma‐Aldrich) for 30 min at 37°C. Subsequently, the coverslips were incubated for 30 min at 37°C with a 1:1,000 dilution of an antivimentin antibody (VI‐10; ab20346 Abcam plc, Cambridge, UK) diluted in PBS containing 0.1% bovine serum albumin. Coverslips were then washed three times with PBS and incubated for 30 min at 37°C with the secondary antibody, an Alexa Fluor 594 anti‐mouse IgG antibody (A11005; Thermo Fisher Scientific). Nuclei were stained and mounted with ProLong™ Gold Antifade Mountant with DAPI (P36931; Thermo Fisher Scientific). Fluorescent microscopy was performed on a Nikon TiS microscope (Nikon Instruments, Amsterdam, The Netherlands).

### 2.3 Karyotyping

For cytogenetic analysis, the karyotype with Bands‐G of the fibroblast cell cultures was performed by the Andalusian Public Health System Biobank. To carry out analysis, first, the cells are incubated in medium supplemented with 0.1 mg/ml of colcemide for 4 hr. Later, they are washed with PBS, trypsinized, and centrifuged. The pellet are resuspended in a hypotonic solution of KCl (0.075 mol/L), washed to remove the cytoplasm, and the nuclei are fixed with methanol/acetic in a 3:1 ratio (vol/vol). Finally, the metaphases are resuspended in 1 ml of fixative and are fixed in slides. G bands with trypsin‐Giemsa (GTG) are made, and a minimum of 20 metaphases are analysed for each cell line, assigning a karyotype formula according to the International System of Human Cytogenetic Nomenclature (McGowan‐Jordan, Simons, & Schmid, 2016). The karyotypes are analysed using a microscope Leica DM5500 and the Ikaros Karyotyping System (Metasystems).

### 2.4 Generation of the NFAH pad

**Figure 1:**
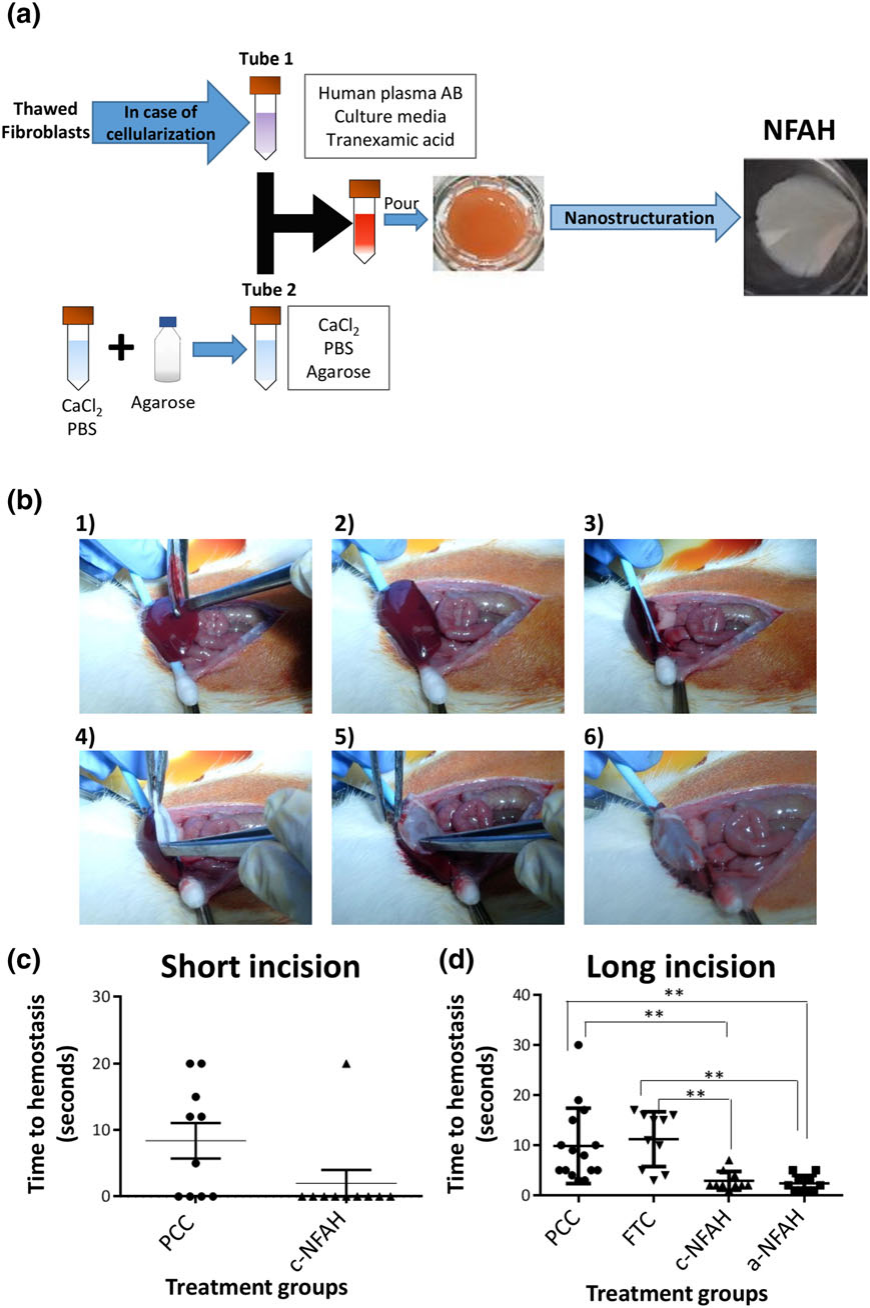
Generation and application of nanostructurated fibrin‐agarose hydrogel (NFAH). (a) Generation scheme of NFAH. (b) Sequential NFAH application on rat hepatic resection. Images show hepatic incision, wound exposition (1–3), NFAH application, and final positioning (4–6). (c,d) Time to haemostasis for rats subjected to (c) short and (d) long hepatic incision treated with different haemostatic agents (*N* = 10). Data represent mean ± *SEM*. Mann–Whitney U test was used for experiments with two groups, whereas the analysis between more than two groups was performed by Kruskal–Wallis analysis of variance test followed by Dunn's post hoc test (**p < 0.01) [Colour figure can be viewed at wileyonlinelibrary.com]

The protocol for NFAH production is an adaptation of methods described previously (Alaminos et al., 2006; Carriel et al., 2012; 2013; 2014; Garzón et al., 2014; Scionti et al., 2014). To obtain the volume of fibrin‐agarose hydrogel (FAH) needed to coat a six‐well plate with 24 mm transwell inserts (3450; Corning Incorporated, Kennebunk, ME, USA), a 30‐ml mixture was prepared as follows: 25 ml of human plasma (Tebu‐bio, Le‐Perray‐en‐Yvelines, France), 0.5 ml of tranexamic acid (Amchafibrin 500 mg, Rottapharm, Milan, Italy), and 2 ml of culture medium, with or without fibroblasts, was placed into a 50 ml conical tube. Subsequently, a solution containing 1.8 ml of 10% calcium chloride (B.Braun, Melsungen, Germany), 1.2‐ml PBS, and 1.5 ml of liquid 2.2% type VII agarose (Sigma‐Aldrich) was added. After mixing, 5 ml of the resulting solution was poured into each transwell, and the plate was left at 37°C for 2 hr. Once gelation was complete, the FAH was covered with culture medium and maintained at 37°C for 24 hr (plates remained in the incubator for a further 7 days when FAH contained cells), prior to nanostructuration (Figure 1a).

Nanostructuration is a compression and dehydration process. This technique preserves the fibrin structure while removing most of its water content. As a consequence, rheological properties of FAH are changed increasing elasticity and resistance (Ionescu et al., 2011). To this purpose, FAH was placed between two extra‐thick western blotting filter paper (88620; Thermo Fisher Scientific). Two 10‐μm nylon net filters (NY‐1009000; Merck Millipore, Burlington, MA) were positioned between the sample and the blotting paper to prevent adherence. Rapidly, a flat glass surface of 0.25 kg is positioned on the top for 1 min and 40 s for compression. The final acellular or cellularized NFAH has a high density with approximately 80% dehydration and a thickness of 50–60 μm.

### 2.5 Animal protocol and hepatic resection

Male Wistar rats (200–250 g) were distributed in two experimental groups, according to the extension of tissue resection. The first short incision or mild experimental model (*n* = 20) includes two animal groups distributed in PCC (*n* = 10) and c‐NFAH (*n* = 10). The second long incision or severe experimental model (*n* = 45) includes four animal groups distributed in PCC (*n* = 15), FTC (*n* = 10), c‐NFAH (*n* = 10), and a‐NFAH (*n* = 10). Anaesthesia was induced by subcutaneous injection of 80‐mg/kg ketamine and 10‐mg/kg xylazine and was maintained by isoflurane inhalation. After abdominal shaving, animals underwent a longitudinal laparotomy 1 cm below the xiphoid process in the craniocaudal direction, leaving the hepatic median lobe exposed for the procedure. Hepatic resection was performed in animals with an incision of 0.5 (short incision or mild model) or 1.5 cm (long incision or severe model) in length.

All haemostatic agents were applied as round‐shaped pads of 12‐mm diameter for the short incision and 24 mm for the long incision. Time to haemostasis was defined as the time needed for the arrest of free blood leakage after application of the haemostatic agent. The timer was started at the moment of application of the haemostatic agent over the hepatic surface and was stopped when a complete cessation of blood extravasation through the hepatic resection surface was achieved.

Immediately postrecovery, rats were housed in individually ventilated cages with free access to food and water. If animals required analgesia, ketoprofen was administrated every 12 hr.

Rats were euthanized by cardiac puncture 24 hr after surgery to evaluate post‐operative haemorrhage, presence of haematoma, migration of the haemostatic agent, and intra‐abdominal adhesion of the haemostatic agent to adjacent intact tissues. Blood was also drawn for the measurement of cytokines by Enzyme‐Linked ImmunoSorbent Assay (ELISA). The grade of adhesion was determined by a score of 0–2: 0, no adhesion; 1, thin adhesions separable by gravity; 2, thick adhesions not separable by gravity.

Sections of hepatic tissue attached to the haemostatic agent were fixed in 4% paraformaldehyde for histopathological analysis.

All procedures were performed according to “Guide for the Care and Use of Laboratory Animals” published by Ministry of Agriculture, Fisheries and Food (R.D. 53/2013, Law 32/2007) and European Communities Council Directive 2010/63/UE. The protocol was approved by the Research Ethics Committee of University Hospital Virgen Macarena and Virgen del Rocío (internal reference: 1131‐N‐15).

### 2.6 ELISA

Blood samples were collected in EDTA tubes (BD Bioscience, NJ, USA), centrifuged at 1,480 × *g* for 5 min and stored at −20°C in small volumes to avoid repeated freeze–thaw cycles. The following commercial ELISA kits were used for cytokine analysis: rat C‐reactive protein (CRP) ELISA Kit (Catalog N° ELR‐CRP, RayBiotech Inc., Norcross, GA, USA), rat interleukin 1 beta (IL‐1β) ELISA Kit (Catalog N° E‐EL‐R0012, Elabscience, Houston, TX, USA), and rat tumour necrosis factor alpha (TNF‐α) ELISA Kit (Catalog N° CSB‐E11987R, Cusabio Technology LLC, Houston, TX, USA).

### 2.7 Microscopy analysis

Paraformaldehyde‐fixed paraffin‐embedded blocks of liver tissue were cut at a thickness of 4 μm, and sections were stained with the Trichrome Stain (Masson) Kit (Catalog N° HT15‐1KT, Sigma‐Aldrich) and haematoxylin–eosin (Catalog N° GHS316 and HT110116, Sigma‐Aldrich), by standard methods. Sectioned liver samples were analysed by investigators blinded to the treatment groups.

Histological variables were studied by microscopic analysis and categorized as shown in Table 1.

**Table 1:** Categorization criteria for histological variables studied by microscopic analysis

Variable	Category			
	Absent	Mild	Moderate	Intense
Haemorrhage	Groups of erythrocytes that come together without forming a free zone of haemorrhage	Area of haemorrhage: <1‐mm diameter	Area of haemorrhage: 1‐to 2.5‐mm diameter and less than ×40 hpf of depth	Area of haemorrhage: >2.5‐mm diameter or more than ×40 hpf of depth
Inflammation	—	Few granulocytes	Some infiltrates, perivascular cuffing	Massive infiltration
Necrosis	—	0.1–0.4 hpf	0.5–1.2 hpf	>1.2 hpf

*Note*. hpf: high‐power fields.

### 2.8 Statistical analysis

Based on our preliminary data, the sample size was calculated to detect a significant effect *d* = 0.55, with a power of 80% at a level of significance of 5%. This requires a minimum of 10 animals per group (https://www.anzmtg.org/stats/PowerCalculator/PowerANOVA).

Data are presented as mean ± *SEM*. Significance was determined using the Mann–Whitney U test or the Kruskal–Wallis analysis of variance test with Dunn's post hoc multiple comparison test. Differences were considered significant at *p* ≤ 0.05. All statistical analyses were performed using GraphPad Prism 7.0 (GraphPad Software Inc., San Diego, CA).

## 3 RESULTS

### 3.1 NFAH has a superior haemostatic effect

NFAH pads comprise a nanostructured fibrin‐type VII agarose hydrogel with (c‐NFAH) or without embedded (a‐NFAH) cells (Figure 1a). To prepare the cellularized solution, we used 150,000 thawed rat fibroblasts (at Passage 2) per millilitre of final volume. Fibroblasts showed robust vimentin expression and a normal karyotype, as expected for healthy proliferating fibroblasts (Figure S1).

We first studied the haemostatic effect of c‐NFAH in a mild model of hepatic resection (short, 0.5‐cm incision). The c‐NFAH pad was prepared as a round‐shaped patch of 12 mm and was placed onto the bleeding surface (Figure 1b). Results showed that the effectiveness of haemostasis was significantly greater with the c‐NFAH pad than with the PCC pad (*p* < 0.05), with a mean time to haemostasis of 2 ± 2 s (*N* = 10, range = 0–20) and 8.4 ± 2.6 s (*N* = 10, range = 5–20), respectively (Figure 1c).

We next tested the haemostatic effect of the c‐NFAH pad in a severe model of hepatic resection (long, 1.5‐cm incision). We also included three additional treatment groups in this analysis: a‐NFAH, to evaluate whether fibroblasts had a beneficial effect on the haemostatic process; FTC, to compare the effect of c‐NFAH with another fibrin‐based commercial haemostatic product; and PCC, to compare the fibrin‐based products with a collagen‐based matrix. Results showed that a‐NFAH presented the lowest time to haemostasis at 2.4 ± 0.58 s (*N* = 10, range = 1–5), followed by c‐NFAH at 2.9 ± 1.72 s (*N* = 10, range = 1–7). Both treatments showed a significantly greater haemostatic effect than PCC (9.8 ± 1.02 s [*N* = 15, range = 3–30]) and FTC (11.2 ± 0.49 s [*N* = 10, range = 3–17]; Figure 1d). There was also a similar time to haemostasis between the mild and severe models for c‐NFAH and PCC treatments (Figure S2A).

Regarding applicability, PCC and FTC were applied by manual pressure as indicated by the manufacturers. By contrast, NFAHs were placed onto the wound without applying any additional pressure (Figure 1b). This application procedure was sufficient to reach haemostasis with NFAH treatment. After 24 hr, no presence of rebleeding was found in any treated group, and only one FTC pad was displaced in one animal.

### 3.2 NFAH has a favourable profile outcome for tissue response and inflammatory features

**Figure 2:**
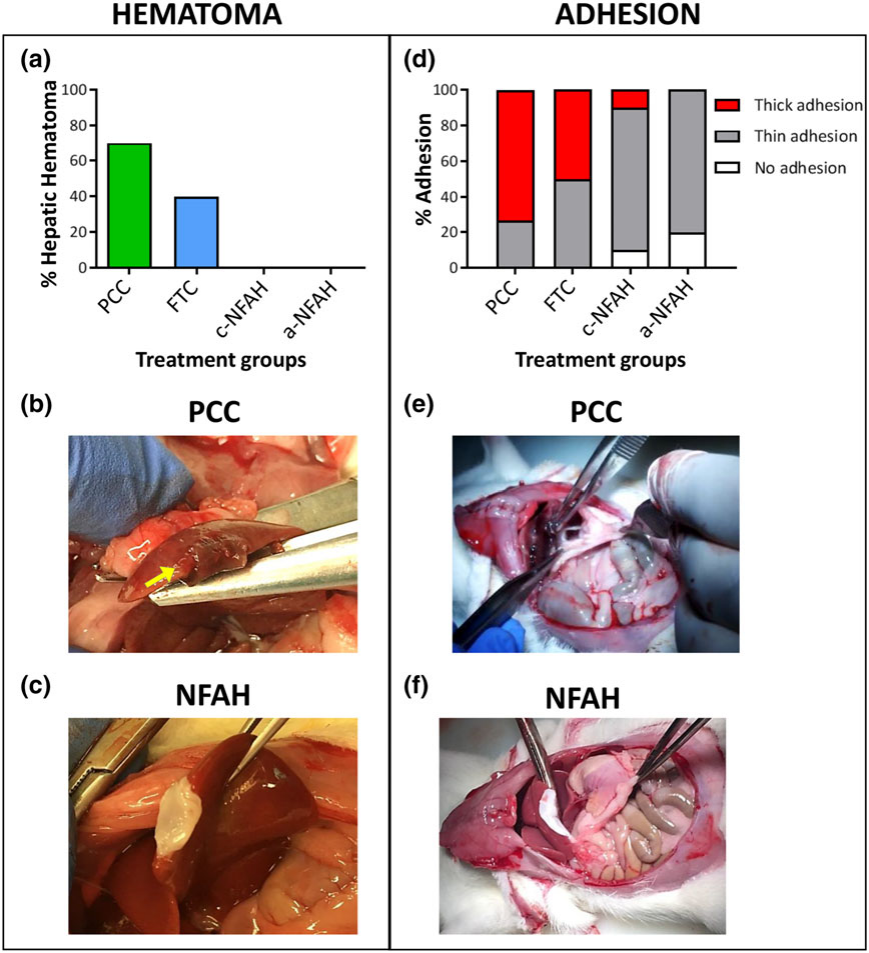
Hepatic haematoma and adhesion grade for different haemostatic agents in rats subjected to long hepatic incisions. (a) Percentage of rats with hepatic haematoma 24 hr after treatment application. (b,c) Representative images of haematoma using PCC (b) and a‐NFAH (c). (d) Adhesion grade of different haemostatic agents. Adhesion in each sample was classified by a score of 0–2: 0, no adhesion; 1, thin adhesions separable by gravity; and 2, thick adhesions not separable by gravity. (e,f) Representative images of adhesion in long hepatic incisions treated with PCC (e) and a‐NFAH (f). c‐NFAH: cellularized‐nanostructurated fibrin‐agarose hydrogel; a‐NFAH: acellular‐nanostructurated fibrin‐agarose hydrogel; FTC: fibrinogen/thrombin‐coated collagen pad (Tachosil®); PCC: protein‐reactive polyethylene glycol‐coated collagen pad (Hemopatch®) [Colour figure can be viewed at wileyonlinelibrary.com]

Visual inspection of the wound 24 hr after the procedure in severe model revealed no incidence of macroscopic haematoma in either c‐NFAH or a‐NFAH groups. By contrast, perilesional haematoma was present in rats treated with PCC and FTC (73.3% and 40%, respectively; Figure 2a). Similar results were obtained in the mild model (Figure S2B). Examples of haematoma with PCC and no haematoma with a‐NFAH are shown in Figure 2b and 2c, respectively.

We also evaluated the presence of undesired adhesions to surrounding healthy tissues at the time of sacrifice. Results showed significant differences among groups, with rats treated with c‐NFAH or a‐NFAH presenting similar results. Remarkably, some of these animals showed no adhesion at all, with most presenting only thin adhesions. As expected, given that PCC an FTC have strong adhesive properties (they have sealant indications), animals treated with PCC and FTC consistently showed thicker adhesion to adjacent organs (Figure 2d). Examples of high‐grade adhesion induced by PCC and no adhesion by a‐NFAH are shown in Figure 2e and 2f, respectively.

**Figure 3:**
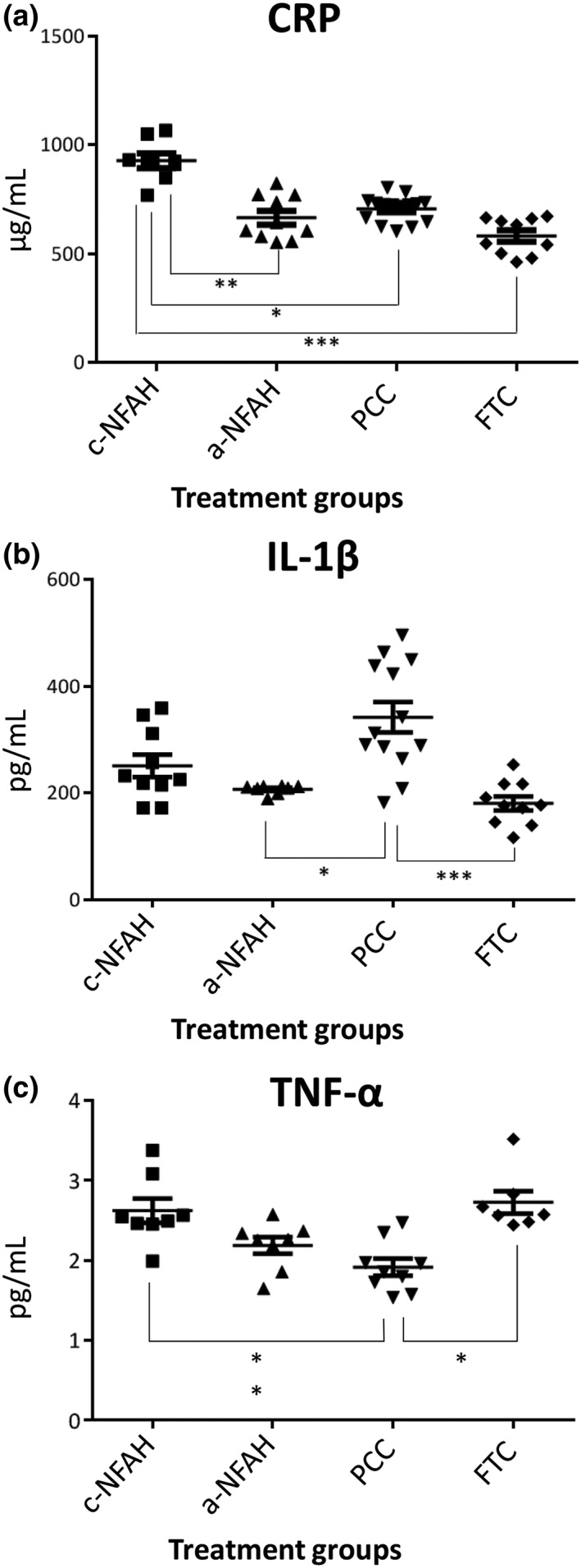
Inflammation factor levels in rats subjected to long hepatic incision after haemostatic treatment. (a) CRP, (b) IL‐1β, and (c) TNF‐α blood levels measured by ELISA after 24 hr in rats with long hepatic incision treated with c‐NFAH, a‐NFAH, PCC, or FTC. Data represent mean ± *SEM*. Data were analysed using Kruskal–Wallis analysis of variance test followed by Dunn's post hoc test (*N* = 7–15); *p < 0.05; **p < 0.01; ***p < 0.001. c‐NFAH: cellularized‐nanostructurated fibrin‐agarose hydrogel; a‐NFAH: acellular‐nanostructurated fibrin‐agarose hydrogel; FTC: fibrinogen/thrombin‐coated collagen pad (Tachosil®); PCC: protein‐reactive polyethylene glycol‐coated collagen pad (Hemopatch®); CRP: C‐reactive protein; IL‐1β: interleukin 1 beta; TNF‐α: tumour necrosis factor alpha

We next measured the levels of inflammatory factors in blood (at sacrifice) by ELISA, to test the inflammatory response at 24 hr following surgery (minimum of *N* = 7 animals per group). Regarding CRP levels, there were no significant differences between the a‐NFAH, PCC, or FTC groups. However, CRP levels in the c‐NFAH group were significantly higher than in the a‐NFAH (*p* < 0.01), PCC (*p* < 0.05), or FTC (*p* < 0.001) groups (Figure 3a). By contrast, the PCC group had significantly higher IL‐1β levels than the a‐NAFH or FTC groups (*p* < 0.05 and *p* < 0.001, respectively), whereas no differences were observed between c‐NAFH, a‐NAFH, and FTC groups (Figure 3b). Furthermore, there were no significant differences in TNF‐α levels between the a‐NAFH group and the other three groups. However, the c‐NAFH and FTC groups presented significantly higher levels than the PCC group (*p* < 0.01 and *p* < 0.05, respectively; Figure 3c).

**Figure 4:**
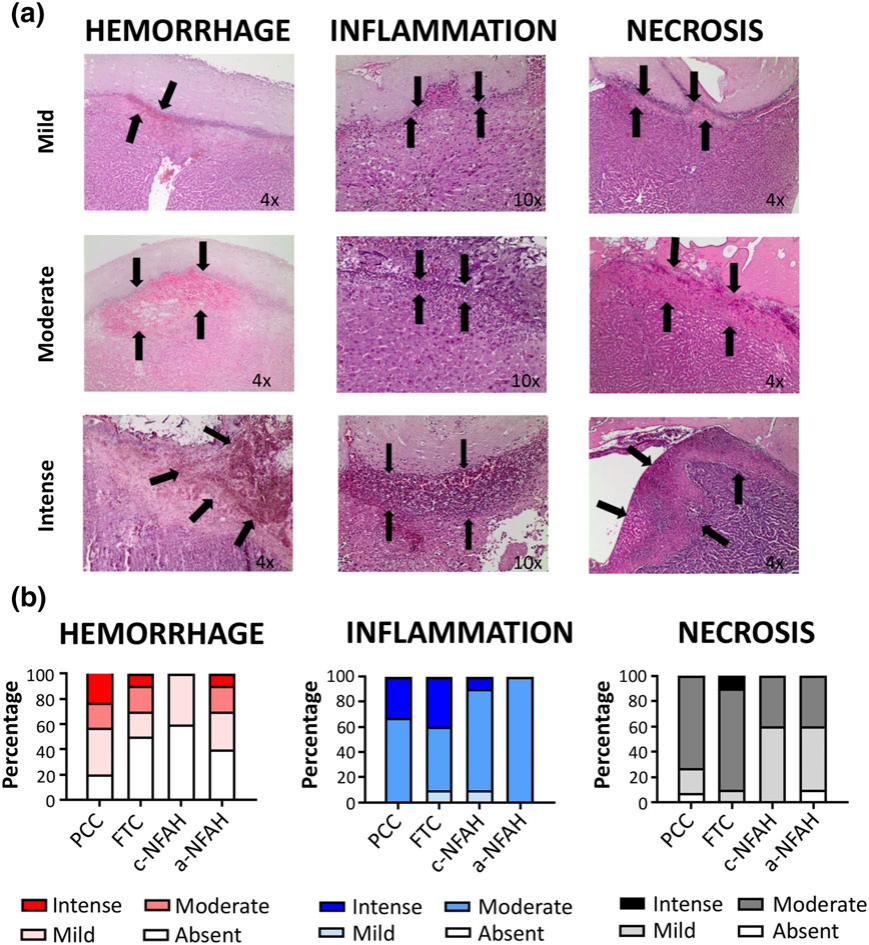
Haemorrhage, inflammation, and necrosis scores in rats subjected to long hepatic incision treated with different haemostatic agents. (a) Representative histological images showing different grades of haemorrhage, inflammation, and necrosis. (b) Percentage of rats with haemorrhage (left), inflammation (middle), and necrosis (right) in long hepatic incision treated with PCC, FTC, c‐NFAH, or a‐NFAH. Each sample was classified as absent, mild, moderate, or intense (see Table 1 for categorization). c‐NFAH: cellularized‐nanostructurated fibrin‐agarose hydrogel; a‐NFAH: acellular‐nanostructurated fibrin‐agarose hydrogel; FTC: fibrinogen/thrombin‐coated collagen pad (Tachosil®); PCC: protein‐reactive polyethylene glycol‐coated collagen pad (Hemopatch®) [Colour figure can be viewed at wileyonlinelibrary.com]

Histopathological variables, including haemorrhage, inflammation, and necrosis, were scored by blinded investigators according to the criteria described in Section 2. Representative images of the different grades of these variables are shown in Figure 4a. Both NFAH treatments showed lower haemorrhage, inflammatory response, and necrosis than PCC or FTC treatment. Further, the PCC group displayed more overall haemorrhage than the other groups. Conversely, the c‐NFAH treatment group presented the lowest percentage of animals with haemorrhage at the microscopic level (40%), showing only mild haemorrhage in some animals (Figure 4b). Moreover, only 10% of the animals in the a‐NFAH group showed intense haemorrhage, which was similar to the FTC group. Concerning inflammation, both NFAH groups had the best profile, with only 10% of animals exhibiting intense inflammation (in the c‐NFAH group). However, both PCC and FTC treatments showed more than 30% of animals with intense inflammation. A similar trend was found for necrosis. Accordingly, the NFAH groups presented the highest percentage of absent and mild necrosis, with no events of intense necrosis, whereas rats treated with PCC and FTC presented a higher percentage of moderate and intense necrosis.

## 4 DISCUSSION

In this study, we demonstrate the haemostatic effectiveness of a novel nanostructured fibrin‐type VII agarose hydrogel, with (c‐NFAH) or without (a‐NFAH) embedded allogeneic fibroblasts. We compared the haemostatic features of NFAH with PCC and FTC, which are widely used in clinical practice. Moreover, we performed a procedure consisting of a short (0.5 cm) or long (1.5 cm) incision to create a mild or severe surgical model of hepatic resection.

In a preliminary mild model study, c‐NFAH showed greater haemostatic ability than the commercially available PCC matrix, prompting us to extend the study to a more severe model of hepatic incision. We also included the a‐NFAH and FTC substrates in the study. a‐NFAH was incorporated to assess whether the presence of fibroblasts provided a significant improvement in haemostasis (Costa‐Almeida, Soares, & Granja, 2017) against the hypothesis that NFAH matrix could account for most of the haemostatic potential of the pads. FTC was incorporated to compare NFAH with a commercial haemostatic agent of similar composition. In this second analysis, c‐NFAH and a‐NFAH showed significantly lower time to haemostasis when compared with PCC and FTC; however, no significant differences were found between c‐NFAH and a‐NFAH. Interestingly, in our hepatic surgical model, better haemostasis was reached using PCC than FTC, in line with a previous report (Lewis et al., 2014).

In addition to time to haemostasis, we investigated other relevant outcomes such as incidence of haematoma formation, rebleeding and/or adhesions, levels of some inflammatory factors, and histology. In contrast to the PCC and FTC treatments, both NFAH treatments showed a complete absence of haematoma formation and a low incidence of thick adhesions. These results might be due to a combination of the fast adherence of the NFAH pads, their lower time to haemostasis and their easier applicability to the wound surface. Contrary to a previous study, PCC had a higher incidence of haematoma formation than FTC in our model (Lewis et al., 2014). Overall, a‐NFAH was superior to the other groups regarding inflammatory factors, with levels equal to or lower than those found with the commercial products. However, c‐NFAH had significant higher CRP levels than a‐NFAH, likely due to the presence of cells. Similarly, NFAH treatment groups had lower grades of haemorrhage, inflammation, and necrosis.

An additional advantage of NFAHs is the smaller adherent effect on surrounding tissue in contrast with hemostats PCC and FTC. This feature can be a great asset to certain types of surgeries.

Our novel NFAH matrix is composed of a nanostructured fibrin‐type VII agarose hydrogel that is completely absorbable and biodegradable and displays optimal biomechanical and rheological properties (flexibility, elasticity, and mechanical strength), with no tissue reactivity or antigenicity (Garzón et al., 2009; Fernández‐Valadés Gámez et al., 2017). Agarose is a polysaccharide extracted from certain algae that, besides being an inert product from an immunological perspective, offers natural adhesiveness. This feature, as well as the high fibrin content (such that it does not rely on fibrinogen conversion as FTC, for example), may contribute to the haemostatic effectiveness of NFAH and the rapid sealing of damaged tissue.

We need to mention that, besides the fibrin, other agents used in the formulation may contribute to the haemostatic effect of our NFAH. Tranexamic acid is a procoagulant drug that has an antifibrinolytic action and has been successfully used to reduce blood loss in several types of surgery (Meng, Pan, Xiong, & Liu, 2018; Queiroz et al., 2018; Suh, Kyung, Han, Cheong, & Lee, 2018; Zilinsky et al., 2019). Indeed, it has been shown that the use of tranexamic acid and fibrinogen reduces blood loss and improves coagulation measurements in a porcine model of liver injury (Zentai et al., 2016). However, the final content of tranexamic acid in the NFAH (1.6% or 8.3 mg) is quite low compared with the concentrations used for topical application (100–500 mg) or intravenously (1 g). Similarly, calcium chloride accelerates fibrin polymerization and clot formation (Brass, Forman, Edwards, & Lindan, 1978), and although it is used to stabilize the matrix, we cannot exclude that the haemostatic effect of the NFAH is enhanced by the presence of calcium.

Furthermore, NFAH has been used as a scaffolding matrix for different artificial tissues, with excellent clinical results in terms of safety thus far (ClinicalTrials.gov: NCT01765244).

The surgical model developed herein may not be absolutely predictive for clinical use. Nevertheless, it does allow a standardized evaluation of our novel hydrogel pad against two commercially available haemostatic agents. As this study was designed as a proof‐of‐concept, we chose rats as our first experimental model, mainly due to their easy availability and handling and low cost. Further investigations are warranted to evaluate NFAH haemostatic effectiveness in other surgical‐based experimental models. Indeed, studies to evaluate long‐term safety and a method of NFAH preservation are currently underway in our laboratory.

As mentioned, no optimal haemostatic agent exists to date, and uncontrolled surgical bleeding remains an unmet clinical need. Any improvement in terms of reduction of time to haemostasis and/or rebleeding, along with ease of use and applicability, could pose a huge impact not only in hospital costs but also in patients' quality of life. We show here that NFAH has excellent haemostatic and sealing properties, and we believe that these results will pave the way for this new product to be part of the arsenal of haemostatic agents in multiple surgical procedures in the future.

## CONFLICT OF INTEREST

M. A. and A. C. are authors of patent for NFAH (N° application Spanish Patent Office: 200930943). R. C. C., B. F. M., J. M., C. C. and M. A. G. B. are authors of a patent application for the use of NFAH as a haemostatic agent (N° application Spanish Patent Office: P201830346). This publication has no other conflicts of interest.

## AUTHOR CONTRIBUTIONS

Rafael Campos‐Cuerva designed the study, performed the experiments (cell culture, immunofluorescence, generation of the NFAH pad), analysed the data, wrote the manuscript, and participated in the revision and final approval of the manuscript. Beatriz Fernández‐Muñoz performed the experiments (cell culture, generation of the NFAH pad) and participated in the revision and final approval of the manuscript. Francisco Farfán López performed the experiments (microscopy analysis) and participated in the revision and final approval of the manuscript. Sheila Pereira Arenas performed the experiments (ELISA, microscopy analysis) and participated in the revision and final approval of the manuscript. Mónica Santos‐González performed the experiments (cell culture, generation of the NFAH pad) and participated in the revision and final approval of the manuscript. Luis Lopez‐Navas analysed the data, wrote the manuscript, and participated in the revision and final approval of the manuscript. Miguel Alaminos designed the study, provided scientific feedback on manuscript, and participated in the revision and final approval of the manuscript. Antonio Campos designed the study, provided scientific feedback on manuscript, and participated in the revision and final approval of the manuscript. Jordi Muntané performed the experiments (surgery, ELISA) and participated in the revision and final approval of the manuscript. Carmen Cepeda‐Franco performed the experiments (surgery), analysed the data, and participated in the revision and final approval of the manuscript. Miguel Ángel Gómez‐Bravo designed the study, performed the experiments (surgery), and participated in the revision and final approval of the manuscript.

## Supporting information


**Figure S1.** **Characterization of rat fibroblasts isolated by collagenase treatment. (A)** Rat fibroblasts cultured after isolation. **(B)** Vimentin expression in cultured rat fibroblasts was determined by immunostaining analysis. HaCat, an immortal keratinocyte cell line, was used as a negative control. **(C)** Karyotype analysis of single cell‐dissociated culture.

**Figure S2**. **Similar time to hemostasis and hematoma presence between the mild and severe models for c‐NFAH and PCC treatments. (A)** Comparison of time to hemostasis for rats subjected to short and long hepatic incision treated with PCC or c‐NFAH. (*N* = 10). **(B)** Percentage of rats subjected to short incision with hepatic hematoma 24 hours after treatment application. Abbreviations: c‐NFAH, cellularized‐nanostructurated fibrin‐agarose hydrogel; PCC, protein‐reactive polyethylene glycol‐coated collagen pad (Hemopatch®).
