# Supplementary figures and images for: Nanostructured fibrin agarose hydrogel as a novel haemostatic agent
Source: J Tissue Eng Regen Med. 2019 Mar 20;13(4):664–73. doi: 10.1002/term.2831 (PMC6594136; doi:10.1002/term.2831)

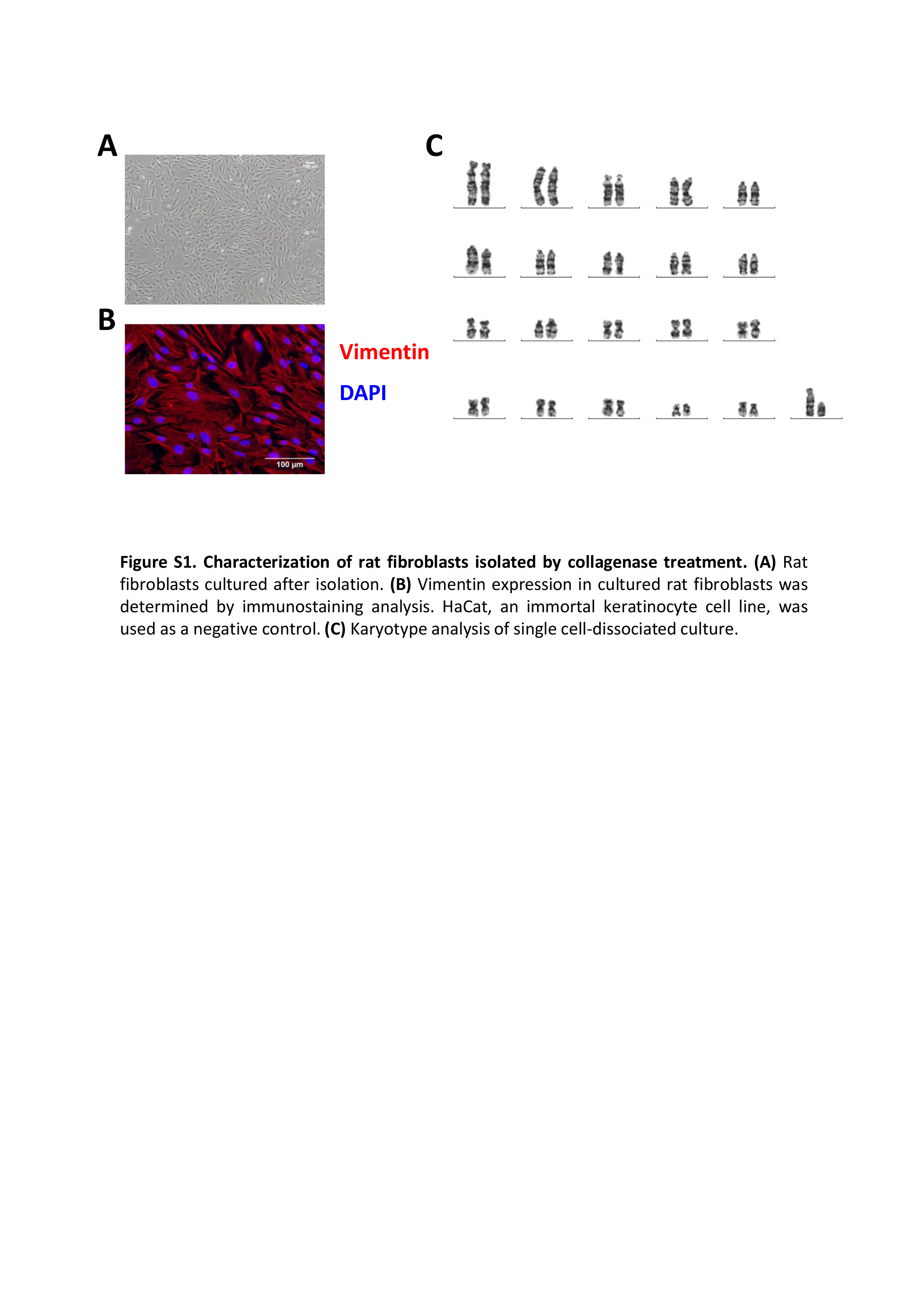

Supplement: Supplementary file 1 — Figure S1. Characterization of rat fibroblasts isolated by collagenase treatment. (A) Rat fibroblasts cultured after isolation. (B) Vimentin expression in cultured rat fibroblasts was determined by immunostaining analysis. HaCat, an immortal keratinocyte cell line, was used as a negative control. (C) Karyotype analysis of single cell‐dissociated culture. [file TERM-13-664-s001.tif]

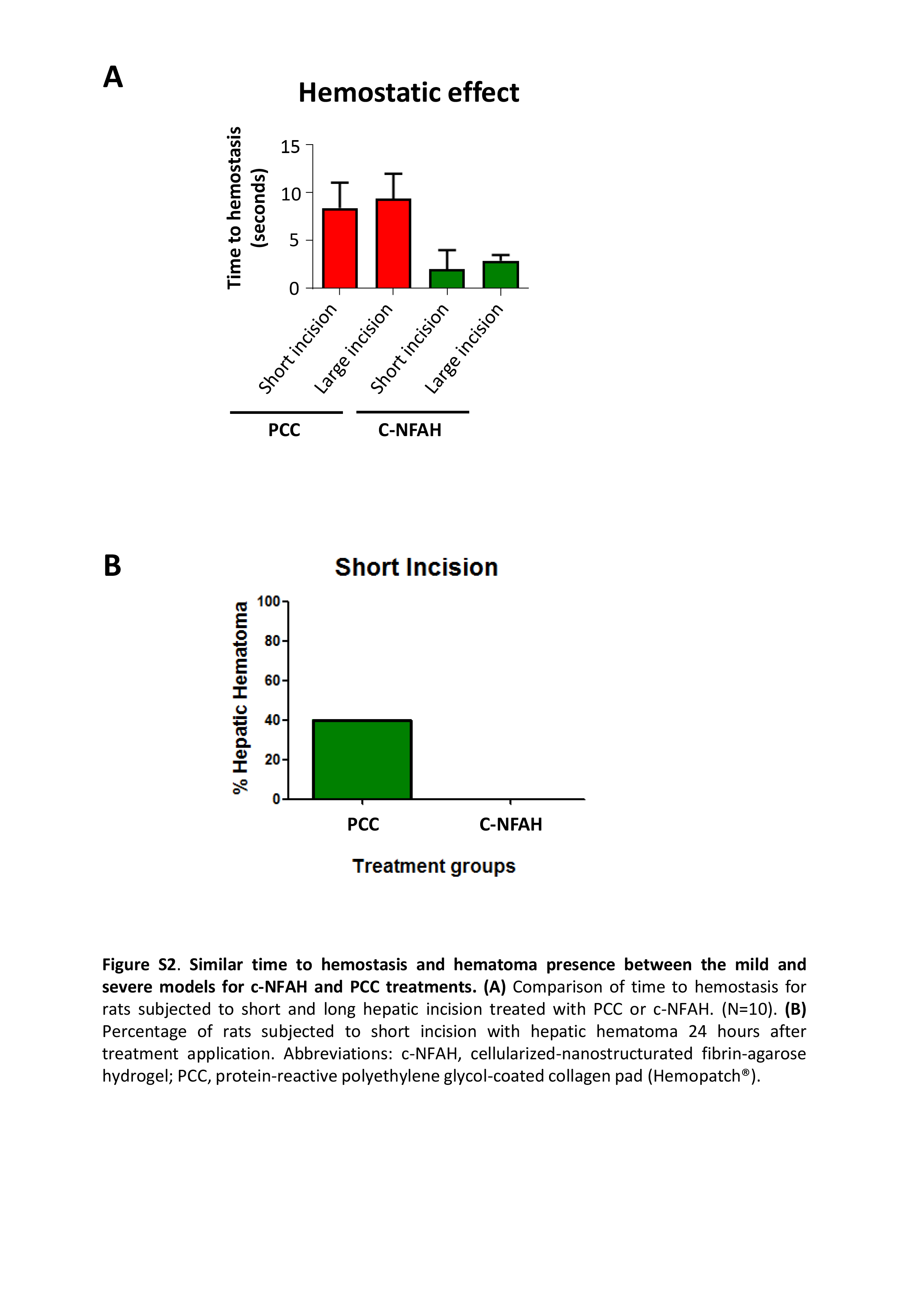

Supplement: Supplementary file 2 — Figure S2. Similar time to hemostasis and hematoma presence between the mild and severe models for c‐NFAH and PCC treatments. (A) Comparison of time to hemostasis for rats subjected to short and long hepatic incision treated with PCC or c‐NFAH. (N = 10). (B) Percentage of rats subjected to short incision with hepatic hematoma 24 hours after treatment application. Abbreviations: c‐NFAH, cellularized‐nanostructurated fibrin‐agarose hydrogel; PCC, protein‐reactive polyethylene glycol‐coated collagen pad (Hemopatch®). [file TERM-13-664-s002.tif]
